# Whole Genome Sequencing and Comparative Genomics of Indian Isolates of Wheat Spot Blotch Pathogen *Bipolaris sorokiniana* Reveals Expansion of Pathogenicity Gene Clusters

**DOI:** 10.3390/pathogens12010001

**Published:** 2022-12-20

**Authors:** Sagar Yadav, Zarrine Raazi, Sheelavanta Matha Shivaraj, Deepika Somani, Ramya Prashant, Abhijeet Kulkarni, Rajeev Kumar, Suma Biradar, Shreenivas Desai, Narendra Kadoo

**Affiliations:** 1 Biochemical Sciences Division, CSIR-National Chemical Laboratory, Pune 411008, India; 2 Academy of Scientific and Innovative Research (AcSIR), Ghaziabad 201002, India; 3 Department of Bioinformatics, Savitribai Phule Pune University, Pune 411007, India; 4 Department of Agricultural Biotechnology and Molecular Biology, Dr. Rajendra Prasad Central Agricultural University, Pusa, Samastipur 848125, India; 5 Department of Genetics and Plant Breeding, University of Agricultural Sciences, Dharwad 580005, India

**Keywords:** *Bipolaris sorokiniana*, CAZyme, comparative genomics, spot blotch, whole genome sequencing

## Abstract

Spot blotch is a highly destructive disease in wheat caused by the fungal pathogen *Bipolaris sorokiniana* (teleomorph, *Cochliobolus sativus*). It is prevalent in warm and humid areas, including Africa, Asia, Latin America, and the USA. In the present study, twelve isolates of *B. sorokiniana* were collected from wheat fields in three different geographical locations in India. The pathogenicity of seven sporulating isolates was assessed on ‘DDK 1025’, a spot blotch-susceptible wheat variety under greenhouse conditions. The isolate ‘D2’ illustrated the highest virulence, followed by ‘SI’ and ‘BS52’. These three isolates were sequenced using the Illumina HiSeq1000 platform. The estimated genome sizes of the isolates BS52, D2, and SI were 35.19 MB, 39.32 MB, and 32.76 MB, with GC contents of 48.48%, 50.43%, and 49.42%, respectively. The numbers of pathogenicity genes identified in BS52, D2, and SI isolates were 2015, 2476, and 2018, respectively. Notably, the isolate D2 exhibited a relatively larger genome with expanded arsenals of Biosynthetic Gene Clusters (BGCs), CAZymes, secretome, and pathogenicity genes, which could have contributed to its higher virulence among the tested isolates. This study provides the first comparative genome analysis of the Indian isolates of *B. sorokiniana* using whole genome sequencing.

## 1. Introduction

Wheat, rice, and maize form an essential food component worldwide and account for approximately 80% of total food grains production in the world. Wheat is the second most widely grown and consumed food crop globally after rice and is the staple food of approximately 35% of the world’s population. Current global wheat production is about 760.9 million tons (https://www.fao.org/faostat/en/#data/QCL; accessed on 3 September 2022). To feed the ever-increasing population of the world from diminishing agricultural land, it is essential to effectively manage the biotic stresses affecting wheat [1]. The major fungal diseases of wheat include rusts, fusarium head blight, powdery mildew, spot blotch, *Septoria tritici* blotch, tan spot, and glume blotch [2,3].

Spot blotch is a foliar disease caused by the air and soil-borne fungal pathogen *Bipolaris sorokiniana*, the anamorph (asexual stage) of *Cochliobolus sativus* (teleomorph, sexual stage). The estimates of yield losses in wheat due to spot blotch vary from 15.5 to 19.6% [4], 20 to 80% [5], and may reach up to 100% under severe infection conditions [6]. Losses of up to 85% in Zambia [7] and 40% in the Philippines [8] have been reported. In highly susceptible wheat varieties, Hetzler et al. [9] reported yield losses of up to 87% due to *B. sorokiniana*. It is the predominant pathogen of foliar blight disease in the Indian states of Bihar, Delhi, Gujarat, Haryana, Karnataka, Maharashtra, Rajasthan, Uttar Pradesh, West Bengal, as well as in the neighboring countries of Bangladesh and Nepal [10].

*B. sorokiniana* isolates vary considerably in their morphology, pathogenicity, and virulence. Variation in pathogenicity of *B. sorokiniana* under different conditions or locations was reported from Pakistan and Nepal [11,12]. Pandey et al. [13] employed random amplified polymorphic DNA (RAPD) markers to identify pathogen isolates belonging to different groups based on their morphological variation. The authors recommended that the genes for developing resistant cultivars should be selected according to the virulence genes present in pathogen isolates prevalent in a particular geographical region. This selection requires the information about the pathogen population structure in a specific area, which would differ in various regions with respect to different virulence genes [14].

Over the past ten years, advances in Next Generation Sequencing (NGS) technologies and decreasing sequencing costs have led to an increase in the sequencing of several plant pathogenic fungi hampering crop production. Sequencing pathogenic fungi helps in exploring the host–pathogen interactions at the molecular level in several pathosystems and leads to the identification of several genes and virulence factors that play essential roles in infection and disease progression [15,16]. The whole genome sequence of several *Bipolaris* species belonging to different species complexes, viz., *Bipolaris sorokiniana, B. maydis, B. oryzae, B. zeicola, B. cookei,* and *B. victoriae,* are publicly available, providing an impetus to the *Bipolaris* research. The comparative genomics of important gene classes among different *Bipolaris* species and related fungi can help reveal the core genes in the genus *Bipolaris* and the expansion and deletion of vital gene classes attributed to their specific lifestyles.

Till now, whole-genome sequences of eight *B. sorokiniana* isolates are available. The genome size of the *B. sorokiniana* strain ND90Pr is 34.42 Mb with 12,250 genes. In ND90Pr and ND93-1 isolates, 60,448 SNPs and 121 polymorphic SSRs were detected. The genes encoding enzymes such as NRPSs and PKSs involved in synthesizing several secondary metabolites, which control virulence, have been identified. Some of these genes are unique and conserved in *B. sorokiniana* [17,18]. Recently, Aggarwal et al. [19] announced the whole genome of the *B. sorokiniana* strain BS112, an Indian isolate (RCTM00000000) with a genome assembly size of 35.64 Mb. This whole-genome de novo sequence represents the first Indian genome-scale assembly for *B. sorokiniana*. However, detailed sequence analysis regarding pathogenicity-related genes, carbohydrate-active enzymes, and comparative genome analysis with other isolates is not available.

In the present study, we collected 12 *B. sorokiniana* isolates exhibiting high morphological variations from wheat fields in three different geographical locations of India and evaluated their pathogenicity on a spot blotch-susceptible wheat variety under greenhouse conditions. The three most virulent isolates were identified and sequenced. Comparative genomic analysis was performed to understand the factors governing the differences in their aggressiveness in causing the spot blotch disease. Interestingly, the isolate D2 had a relatively larger genome with an expanded set of pathogenicity-related genes, which might contribute to its higher virulence. The study provides the pathogen genomic resources for further identification and characterization of key pathogenesis-related genes and would aid in the development of better disease control measures.

## 2. Materials and Methods

### 2.1. Collection of Isolates

Spot blotch-infected wheat leaves were collected from the fields of Dr. Rajendra Prasad Central Agricultural University (RPCAU), Pusa (Bihar, India), and the University of Agricultural Sciences (UAS), Dharwad (Karnataka, India). The collected leaves were brought to the laboratory and surface-sterilized with 2% sodium hypochlorite (NaOCl) (HiMedia, Mumbai, India). Approximately 1 sq. cm leaf pieces containing spot blotch patches were transferred to plates containing potato dextrose agar (PDA) (HiMedia, Mumbai, India). The PDA plates were incubated at 28 °C with a 12 h photoperiod for seven days. A total of 11 isolates were obtained. The isolates D2, HD3069, A, L, O1, O2, N1, Bp, and J were obtained from spot blotch-infected wheat leaves collected from RPCAU, Pusa (Bihar, India), while the isolates DI and SI were obtained from UAS, Dharwad (Karnataka, India). The isolate BS52 was provided by Dr. Rashmi Aggarwal, ICAR-Indian Agricultural Research Institute, New Delhi, India, which had been collected from the fields of Shillongani, Assam, India [20]. The 12 isolates were again grown separately on PDA plates, and the plates were regularly observed under a stereo-zoom microscope (Leica, Wetzlar, Germany) at 40X magnification for sporulation. The fungal isolates were deposited in the National Fungal Culture Collection of India (NFCCI), Agharkar Research Institute, Pune, India under the accession numbers NFCCI5337– NFCCI5348.

### 2.2. Establishment of Monoconidial Cultures

Only 7 isolates (A, J, L, HD3069, BS52, D2, and SI) sporulated under the laboratory conditions. Multiple attempts to induce sporulation in other isolates using various growth media and incubation conditions were unsuccessful. Hence, single spore (monoconidial) cultures could be established only for the 7 isolates using the following procedure. Conidia from each isolate were harvested by scraping the surface of the PDA plates with an inoculation loop and suspended in sterile distilled water. The suspension was filtered through a sterile muslin cloth to separate the mycelia and spores. Spore count of the suspension was estimated using a hemocytometer. The conidial suspension was diluted to achieve a spore count of approximately 50 spores/mL. Approximately 100 μL of the conidial suspension was plated on water-agar plates containing 150 mg/L of streptomycin and the plates were incubated at 28 °C for 24 h. Spore germination was observed under a stereo-zoom microscope at 80× magnification. Agar discs containing a single germinating spore were lifted using a sterile 5 mm cork borer and transferred to new PDA plates containing 150 mg/L of streptomycin. The plates were incubated at 28 °C with 12 h of light and 12 h of dark for 72 h. This process was repeated twice to purify and establish the monoconidial cultures. Further sub-culturing was performed on PDA plates, and the cultural characteristics of the monoconidial isolates were monitored visually, while the spores were visualized using the stereo-zoom microscope.

### 2.3. Internal Transcribed Spacer (ITS) Sequencing

All the monoconidial cultures were grown in triplicate in Potato Dextrose Broth (PDB) (HiMedia, Mumbai, India) at 28 °C in a shaking incubator with 180 rpm and a 12 h photoperiod. After five days, the mycelia were harvested, crushed, and DNA was isolated using the Qiagen Miniprep DNA isolation kit (Qiagen, Hilden, Germany). The extracted DNA was quantified using a Nanodrop ND-1000 spectrophotometer (Thermo Fisher Scientific, Waltham, MA, USA). Genomic DNAs from all the monoconidial cultures were subjected to PCR using the internal transcribed spacer (ITS) primers ITS1 (5′TCCGTAGGTGAACCTGCGG3′) and ITS4 (5′TCCTCCGCTTATTGATATGC3′) [21]. The amplified products were sequenced using Sanger sequencing and the isolates were confirmed to be *B. sorokiniana* by analyzing the sequences using the Unite ITS database (https://unite.ut.ee/; accessed on 26 May 2021).

### 2.4. Fungal Pathogenicity Assay by Seed Inoculation Method

Pathogenicity of the seven sporulating isolates of *B. sorokiniana* was assessed using a spot blotch-susceptible wheat variety ‘DDK 1025’ [22]. For pathogenicity assay, the seed inoculation method as described by Minotto et al. [23] was followed with some modifications. In brief, the seeds were surface-sterilized using 1% sodium hypochlorite (NaOCl) for 5 min and washed thrice with sterile distilled water. The disinfected seeds were placed on moist autoclaved blotting paper in Petri plates, wrapped in aluminum foil, and incubated at room temperature for 48 h for germination. Seeds with aborted germination were discarded and the healthy germinated seeds were inoculated by transferring them to vials containing spore suspension of *B. sorokiniana* isolates. The spore suspension was prepared from seven-day-grown culture of *B. sorokiniana* isolates. Spore concentration was adjusted to 2 × 10^3^ spores/mL of sterile distilled water with 0.1% Tween-20. The inoculated seeds were incubated at 28 °C for 48 h. After incubation, the seeds were washed with sterile distilled water to remove any spores loosely adhering to the seed surface. The seedlings were transferred to surface-sterilized seedling trays filled with autoclaved soil-rite (mixture of 75% Irish peatmoss and 25% horticulture grade expanded perlite) in a greenhouse with a day-time temperature of 28 °C (12 h) and a night-time temperature of 24 °C (12 h) and relative humidity of >90%. The seedlings were regularly monitored for ten days for disease symptoms. Healthy germinated seeds incubated in sterile distilled water containing 0.1% Tween-20 without spore suspension were considered as controls. The three most virulent isolates obtained from this assay were used for further experiments.

### 2.5. Fungal Pathogenicity Assay by Spray Inoculation Method

Based on the seed inoculation assay, the three most virulent *B. sorokiniana* isolates were further assessed for pathogenicity by the spray inoculation method as described by Francesconi et al. [24] with some modifications. The seeds of the spot blotch-susceptible variety ‘DDK 1025’ were surface-sterilized with 1% NaOCl and were imbibed overnight in sterile distilled water followed by in vitro germination under aseptic conditions. The germinated seeds were transferred to surface-sterilized seedling trays and grown in a glasshouse for ten days with a temperature of 28 °C during the day (12 h) and 24 °C during the night (12 h). Spore suspensions were prepared from seven-day-old PDA-grown cultures of *B. sorokiniana* isolates with a spore concentration of 2 × 10^3^ spores/mL. Tween-20 (0.02%) was added as a surfactant. Spore suspension of each isolate was sprayed on 100 plants, divided into 10 replicates with 10 plants in each replicate. After spray-inoculation, the plants were transferred to a humid chamber with a relative humidity of >90% and other conditions as mentioned before. A completely randomized design was used to place the labeled seedling trays within the chambers. The seedlings were monitored daily for disease symptoms. Disease severity was assessed seven days post-inoculation on the second leaf, using the system described by Adlakha et al. [25]. The disease on each plant was scored on a scale from 0 (no leaf spots or chlorosis) to 5 (necrotic spots with chlorosis covering more than 60% of leaf area). Statistical tests were performed using the OPSTAT software (http://14.139.232.166/opstat/default.asp; accessed on 16 November 2021).

### 2.6. DNA Isolation

Monoconidial cultures of the three isolates, BS52, D2, and SI, were grown in Potato Dextrose Broth (PDB) at 28 °C in a shaking incubator with 180 rpm and a 12 h photoperiod for five days. The mycelia were harvested and crushed in liquid nitrogen. DNA isolation was performed using the Qiagen Miniprep DNA isolation kit (Qiagen, Hilden, Germany) and the quality and concentration of the extracted DNA were determined using a Nanodrop ND-1000 spectrophotometer (Thermo Fisher Scientific, Waltham, MA, USA).

### 2.7. DNA Library Preparation and Genome Sequencing

DNA libraries of the three isolates, BS52, D2, and SI, were prepared with 1 µg of DNA using a TrueSeq DNA sample preparation kit (Illumina Cat. No. FC-121-2001). The DNA was fragmented using an ultrasonicator S220 (Covaris, Woburn, MA, USA) to obtain an average of 350 bp fragments. This was followed by end repair, A-tailing, ligation with Illumina adapters, size selection, and PCR amplification. The prepared libraries were quantified using Bioanalyzer 2100 (Agilent, Santa Clara, CA, USA) and quantitative PCR (qPCR). The clusters were generated using cBOT, and paired-end sequencing was performed using Illumina HiSeq 1000 platform (CCAMP, Bangalore, India). Sequencing data in the form of 2 × 100 bp paired-end reads were obtained. To improve the quality of the assembly, mate-pair sequencing of the D2 isolate was also performed using Illumina HiSeq 1000 platform (CCAMP, Bangalore, India).

### 2.8. Genome Assembly and Assessment

#### 2.8.1. *De Novo* Assembly

The raw paired-end reads of the three isolates and mate-pair reads of the isolate D2 were subjected to FastQC analysis (https://www.bioinformatics.babraham.ac.uk/projects/fastqc/; accessed on 12 December 2021) to evaluate the quality of reads in terms of the presence of adapter sequences. The NGS QC Toolkit (https://bioinformaticshome.com/tools/rna-seq/descriptions/NGS_QC_Toolkit.html; accessed on 15 December 2021) was used for quality control and data filtration. The resulting high-quality paired-end reads (for all three isolates) and mate-pair reads (for D2 only) were provided as input to the ALLPATHS-LG (http://software.broadinstitute.org/allpaths-lg/blog/; accessed on 16 December 2021) whole-genome shotgun assembler [26]. The assembly was performed using the default k-mer value of 96. The final assembly using ALLPATHS-LG had a higher N50 value, a smaller number of contigs, and less N contamination with a fair value of final assembly. The *Cochliobolus sativus* ND90Pr genome (https://www.ncbi.nlm.nih.gov/Taxonomy/Browser/wwwtax.cgi?id=665912; accessed on 20 December 2021) comprising 157 scaffolds with a genome size of 34.42 MB was used as the reference for assembly. Assemblies with the best combination of high sequence coverage, N50 and L50 values, and the least number of contigs were selected as the best representative assembly for a particular isolate.

#### 2.8.2. Assessment of Genome Completeness and Phylogenetic Analysis

The genome assembly was validated using BUSCO (Benchmarking Universal Single-Copy Orthologs; https://busco.ezlab.org/; accessed on 23 December 2021) (v2) [27]. Eleven genome sequences, including those of various *Bipolaris* species (*B. oryzae* TG12bL2, *B. oryzae* ATCC 44560, *B. victoriae* Fi3, *B. zeicola* 26-R-13, *B. maydis* C5, *B. maydis* ATCC 48331, and *B. cookei* LSLP18.3) and four *B. sorokiniana* strains (*B. sorokiniana* BS112, *B. sorokiniana* WAI2411, *B. sorokiniana* ND90Pr, and *B. sorokiniana* BRIP27492a), were downloaded from NCBI (https://www.ncbi.nlm.nih.gov/; accessed on 23 December 2021). These eleven genome sequences, along with the assembled genomes of the three isolates (BS52, D2, and SI), were submitted to BUSCO to evaluate the genome completeness of the three *B. sorokiniana* isolates.

The phylogeny of the three isolates and the other publicly available genome sequences of eleven *Bipolaris* strains was analyzed. The single-copy BUSCO genes were extracted from all fourteen strains/isolates. These genes were individually aligned using MAFFT (v7.305) [28], and the alignments were filtered with trimAl (v1.4) [29]. All the alignments were concatenated, and the maximum likelihood tree was produced using RAxML (v8.1.2) [30]. The resulting tree was rooted using Newick utilities (v1.6) [31].

#### 2.8.3. Genome Synteny

Synteny analysis was performed by comparing the genomes of the three *B. sorokiniana* isolates (BS52, D2, and SI) with the reference genome (*B. sorokiniana* North American isolate ND90Pr; NCBI:txid665912). The syntenic regions were predicted using the MUMmer 3.0 package (http://mummer.sourceforge.net/; accessed on 4 January 2022) [32]. The alignments of scaffold sequences were generated from the NUCmer script present in the MUMmer package using default parameters. The generated data file containing alignments was filtered to retain only the alignments with sequence identity above 99% and length above 10000 bp. The synteny was visualized using Circos (http://circos.ca/; accessed on 5 January 2022) [33]. The total length of syntenic regions was estimated by adding all individual alignments. The percent identity was calculated by dividing the size of the syntenic region by the size of the reference genome, and the product was multiplied by 100.

#### 2.8.4. Gene Prediction and Annotation

Gene prediction was performed using AUGUSTUS (v3.3.2) (http://augustus.gobics.de/; accessed on 8 January 2022) [34] with *Cochliobolus sativus* ND90Pr as the reference. The *C. sativus* ND90Pr coding sequence was downloaded from NCBI (https://www.ncbi.nlm.nih.gov/; accessed on 8 January 2022) and split into training and test data sets. AUGUSTUS was trained using ND90Pr as a training model through which genes were predicted. Functional annotation of the genes was performed by determining homology with *B. sorokiniana* protein sequences in Swiss-Prot (http://www.uniprot.org/; accessed on 9 January 2022) using BLASTP with an e-value of <1e^−5^ and identity greater than 25% (https://blast.ncbi.nlm.nih.gov/Blast.cgi; accessed on 9 January 2022). The annotation of protein domain structures was performed using InterProScan5 [35].

The identification, comparison, and visualization of orthologous gene clusters among the genomes of the *B. sorokiniana* strain ND90Pr and the three isolates BS52, D2, and SI, were performed using the OrthoVenn2 (https://orthovenn2.bioinfotoolkits.net; accessed on 10 January 2022) [36] web platform. The platform utilizes a modified OrthoMCL algorithm to identify the orthologous gene clusters from the UniProt/Swiss-Prot database. The protein sequences for each sample were submitted to EggNOG v5.0 (http://eggnog.embl.de/; accessed on 15 January 2022) [37] to predict the Category of Orthologous Groups (COGs). The scaffold sequences of *B. sorokiniana* isolates BS52, D2, and SI were further analyzed for secondary metabolites gene clusters using antiSMASH v5.0 (https://fungismash.secondarymetabolites.org/; accessed on 21 January 2022) [38]. Further, the protein sequences of *B. sorokiniana* isolates BS52, D2, and SI were subjected to CAZy (Carbohydrate-Active Enzyme) annotation (http://www.cazy.org/Welcome-to-the-Carbohydrate-Active.html/; accessed on 22 January 2022) [39]. SignalP 6.0 (https://services.healthtech.dtu.dk/service.php?SignalP; accessed on 30 January 2022) [40] was used to predict the presence of signal peptides in the protein sequences. Protein sequences of the pathogenicity genes were retrieved from the Pathogen–Host Interaction (PHI) database (http://www.phi-base.org/; accessed on 24 January 2022) [41], and BLASTP analysis was performed against the *B. sorokiniana* proteome. Protein alignments with e value of <1e^−5^ and identity of >25% were considered as putative pathogenicity genes in *B. sorokiniana*. Venn diagrams depicting the number of common genes identified by CAZy, secretory protein, and PHI analyses in BS52, D2, and SI were drawn using the Venn diagram tool available at (https://bioinformatics.psb.ugent.be/webtools/Venn/; accessed on 6 February 2022). The total number of PHI hits used for the Venn diagram included 1783, 2226, and 1788 hits in BS52, D2, and SI, respectively. Further, a Venn diagram for the unique genes found in isolate D2 was drawn, in which the total number of 79, 455, and 410 unique genes showing hits for CAZy, PHI, and secretory proteins, respectively, were depicted.

## 3. Results

### 3.1. Cultural Characteristics of the Pathogen Isolates

Cultural characters of the 12 *B. sorokiniana* isolates varied greatly. The mycelial morphology varied from cottony white to dark (melanized) on the PDA medium (Figure S1). The cultures were also observed under a compound light microscope (Leica, Germany) to reveal their morphological features and culture characteristics. The isolate Bp showed cottony growth with orange pigmentation at the hyphal base, while the isolate HD3069 showed cottony mycelia having a melanized base with a compact bunch of erect mycelia. Although the hyphal growth was a white mass in O1, some melanization was observed at the center of growth. The isolate L showed fruiting structures dispersed through the radial mat. The isolate J showed white mat growth with fur-like mycelium, while the isolate N1 had a dark gray cottony erect mycelial mat. The isolate DI showed the highest melanization and, hence, appeared black on PDA. A mixture of white and olive green-colored hyphae was observed in isolate D2 along with some white fruiting structures. In the O2 isolate, woolly growth was observed, and the hyphae appeared to be more rigid compared to O1. Isolate A showed irregular radial growth and light olive green-colored hyphae were seen with little melanization. A cottony hyphal radial mat was observed in the SI isolate with varied levels of melanization; a white woolly mycelial mat was observed at the periphery in isolate BS52.

### 3.2. Establishment of Monoconidial Cultures

**Figure 1:**
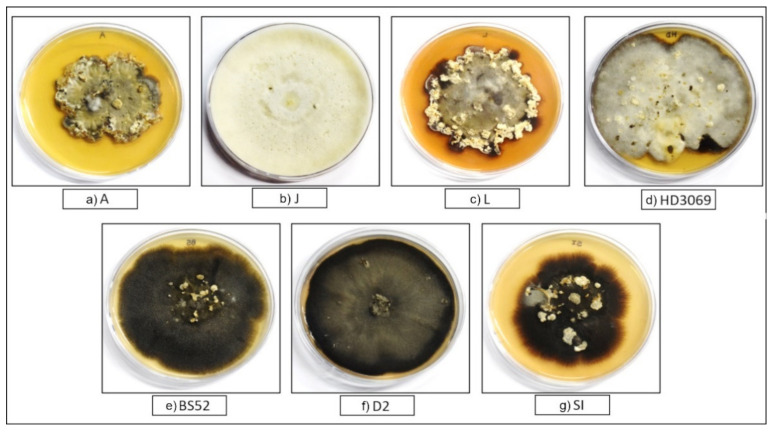
Monoconidial cultures of seven isolates of *B. sorokiniana* (**a**–**g**) grown on PDA plates. The names of the seven isolates are mentioned in capital letters.

As some isolates showed heterogeneous spore morphology under light microscope, it was decided to establish single spore (monoconidial) cultures for all the twelve isolates. However, only seven isolates (A, J, L, HD3069, BS52, D2, and SI) sporulated under the laboratory conditions even after multiple attempts using various media and experimental conditions. Hence, monoconidial cultures could be established only for these seven isolates. The monoconidial cultures showed slow and restricted growth in isolates A and L with dense fruiting structures. White, yellow fruiting bodies were observed in isolate HD3069. White cottony hyphae were observed in isolate J. A high level of melanization was seen in BS52 and D2. The isolate SI showed mixed (white and melanized) types of mycelia with white-colored fruiting bodies (Figure 1).

### 3.3. Pathogen Characterization by ITS Sequencing

To confirm the identity and estimate the genetic variation among the isolates, the ITS region of the *B. sorokiniana* isolates was amplified using ITS1 and ITS4 primers. Amplicons from the seven isolates were sequenced with five replicates each (total 35 samples). For each isolate, all the replicates yielded an identical sequence, confirming that there was no variation among the replicates of each isolate. The BLAST searches revealed homology to the *C. sativus* isolate D_D44. The ITS sequences of all the isolates of *B. sorokiniana* have been deposited in the NCBI database (Accession nos: KJ562714-KJ562718, OP524195 and OP537184).

### 3.4. Spore Morphology

**Figure 2:**
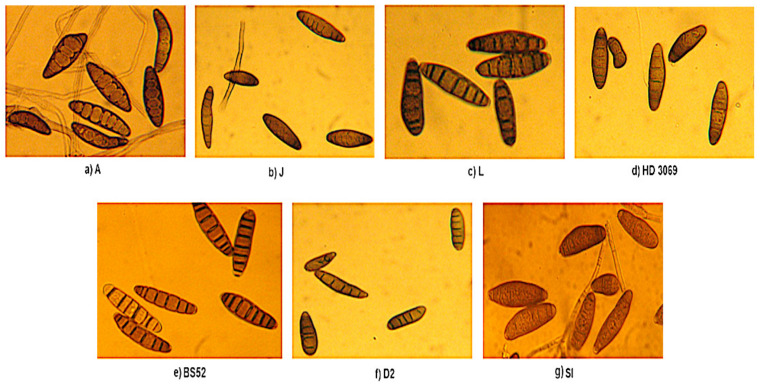
Spores from monoconidial cultures of the seven isolates (**a**–**g**) observed under a compound light microscope. The names of the isolates are mentioned in capital letters.

Between 50 and 100 spores from individual monoconidial cultures of the seven isolates were analyzed for spore morphology using a compound light microscope (Leica, Germany). The conidia varied in size and consisted of between 2 and 13 septa. The conidia from all the isolates were slightly curvaceous, spindle-shaped, and olive green to brown (Figure 2).

### 3.5. Fungal Pathogenicity Assay by Seed Inoculation Method

The mortality rate of seedlings ten days after inoculation was considered to assess the virulence of the seven isolates. All seven isolates were found to be virulent, and the typical spot blotch symptoms appeared on the inoculated plants. However, the infected plants displayed differences in spot blotch symptoms probably due to variation in the aggressiveness of the isolates. Spot blotch appeared as brown spindle-shaped blotches on the tip and the center of the leaves and, in some cases, on the entire leaf margin. The mortality rate of the plants inoculated with the isolate D2 was the highest, followed by those inoculated with SI and BS52. The mortality rate of the plants inoculated with other isolates was lower (Table S1).

### 3.6. Fungal Pathogenicity Assay by Spray Inoculation Method

**Figure 3:**
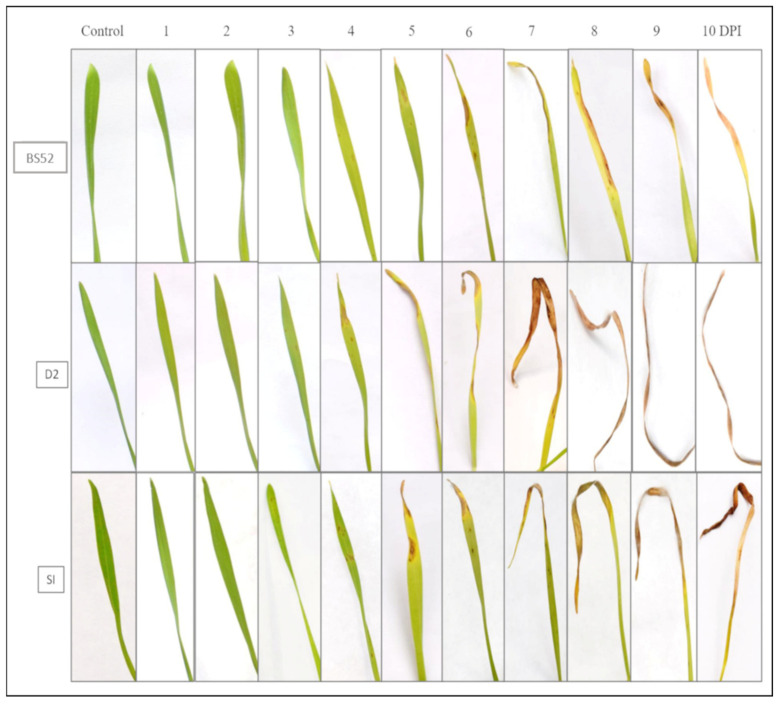
Spot blotch disease symptoms on wheat seedlings on inoculation with three *B. sorokiniana* isolates, BS52, D2, and SI. The numbers at the top indicate the number of days post-inoculation (DPI).

Based on the mortality rate of the plants inoculated with the *B. sorokiniana* isolates by the seed inoculation method, the three most virulent isolates (BS52, D2, and SI) were selected for detailed analyses. Wheat seedlings were grown as described and inoculated with spore suspensions of the three *B. sorokiniana* isolates. The plants were monitored for ten days for spot blotch symptoms. The first signs of infection appeared on the third day after inoculation in the case of D2 and SI, while in BS52, the signs occurred on the fourth day after inoculation (Figure 3). Gradual increase and merging of spots were seen in all three cases, though the severity of the disease varied among the isolates. The disease was scored on the second leaf of seedlings at seven days post-inoculation (Table S2) to assess the disease severity. The Analysis of Variance (ANOVA) revealed significant differences in disease severity among the three isolates. All mean square estimates of treatments were significant at *p* < 0.05. The disease severity did not differ significantly within the replicates (Table S3A). The average disease severity score for BS52 was 3.26 on the scale proposed by Adlakha et al. [25], with most leaves showing necrotic spots with pronounced chlorosis covering from 21–40% of the leaf area. The average score for D2 was close to the maximum value of 5.00 (4.98), with almost all of the leaves showing merged spots covering more than 60% of the leaf area. For SI, the average score was 4.38, with most leaves showing necrotic spots and chlorosis covering from 41–60% of the leaf area (Table S3B). Overall, D2 caused more severe disease than SI and BS52 under greenhouse conditions.

### 3.7. Genome Assembly and Assessment

#### 3.7.1. *De Novo* Assembly

The NGS QC toolkit was used for quality control and data filtration. The filtered number of reads obtained were 41 million, 51 million, and 48 million for BS52, D2, and SI, respectively, for the paired-end data and 256 million for the mate-pair data for isolate D2, which corresponded to genome sequencing depths of 58×, 64×, and 74× for BS52, D2, and SI, respectively, for the paired-end data and 326× for the mate-pair data for the isolate D2. The resulting high-quality paired-end reads (for all three isolates) and mate-pair reads (for D2 only) were provided as input to the ALLPATHS-LG whole-genome shotgun assembler. The read datasets of each isolate were subjected individually to de novo assembly with a default k-mer value of 96. Assemblies with the best combination in terms of sequence coverage, N50 and L50 values, and the least number of contigs were selected as the best representative assembly for that particular isolate. The assembled genome sizes were 35.19 MB, 39.32 MB, and 32.76 MB for BS52, D2, and SI in the final assembly. The scaffold numbers were 280, 317, and 319 in BS52, D2, and SI, with N50 lengths of 1054399, 803483, and 1063709, respectively (Table 1).

#### 3.7.2. Assessment of Genome Completeness

The BUSCO alignment suggested that all three genome assemblies were of high quality, nearly complete, and accurate. Common BUSCOs (3637) were identified in each of the 14 *Bipolaris* genome sequences (11 genome sequences retrieved from NCBI and 3 genome sequences from this study). BUSCO alignment results showed that the final assembly of BS52 contained 6580 complete BUSCOs out of 6641 total BUSCOs (99.08%), of which 6568 were single-copy, while 12 were duplicated. The final assembly of D2 comprised 6579 complete BUSCOs (99.06%), of which 6572 were single-copy, and 7 were duplicated. Similarly, the final assembly of SI contained 6035 complete BUSCOs (90.87%), of which 6030 were single-copy, while 5 were duplicated (Figure S2).

#### 3.7.3. Phylogenetic Analysis

**Figure 4:**
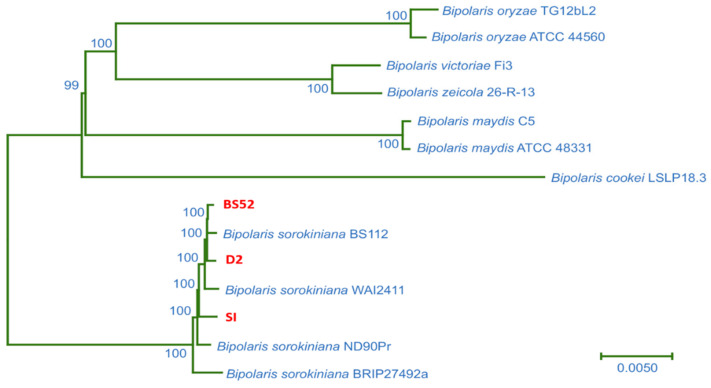
A phylogenetic relation between the three isolates (BS52, D2, and SI) and available *B. sorokiniana* strains and other *Bipolaris* species.

A phylogenetic tree was constructed based on the concatenated alignment of the highly conserved, single-copy orthologs of BS52, D2, and SI, with seven genome sequences from five *Bipolaris* species (*B. oryzae* TG12bL2, *B. oryzae* ATCC 44560, *B. victoriae* Fi3, *B. zeicola* 26-R-13, *B. maydis* C5, *B. maydis* ATCC 48331, and *B. cookei* LSLP18.3) and four genomic sequences from *B. sorokiniana* strains BS112, WAI2411, ND90Pr, and BRIP27492a. The inferred phylogeny showed that the isolates BS52, D2, and SI formed a clade with the four *B. sorokiniana* strains, indicating their genetic relatedness (Figure 4).

#### 3.7.4. Genome Synteny

**Figure 5:**
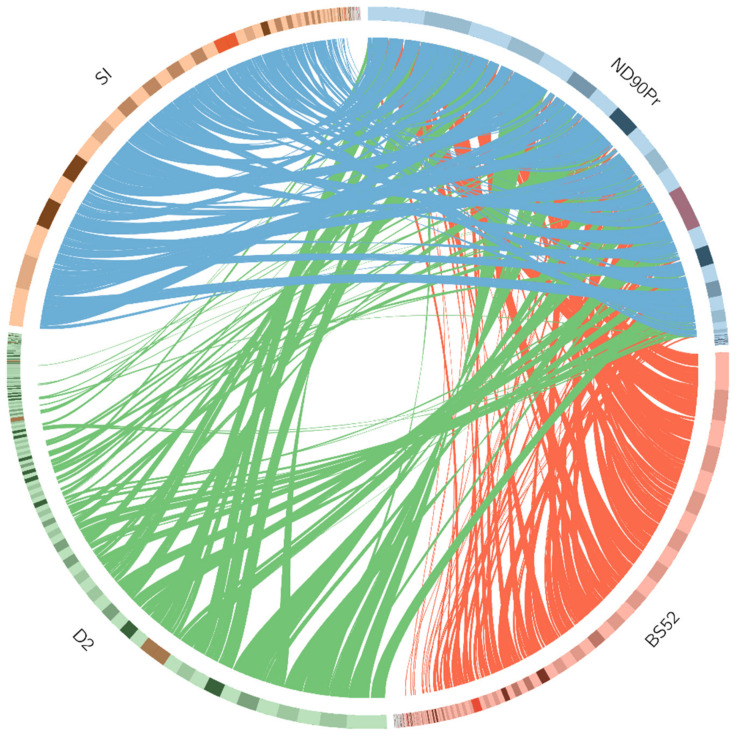
Synteny relationship among the genomes of the *B. sorokiniana* strain ND90Pr and three *B. sorokiniana* isolates (BS52, D2, and SI). The bars in the outer layer represent scaffolds organized from the largest to shortest clockwise. Connected colored lines represent the syntenic relation among ND90Pr and the three isolates.

A genome-wide comparative analysis was performed by comparing the genomes of the three *B. sorokiniana* isolates (BS52, D2, and SI) with the reference genome of the North American *B. sorokiniana* isolate ND90Pr. All three *B. sorokiniana* isolates (BS52, D2, and SI) shared a high degree of genomic synteny with the reference genome ND90Pr (Figure 5). The isolate BS52 shared 85.19% similarity to ND90Pr, while D2 and SI shared 84.78% and 85.04% similarity to ND90Pr, respectively.

### 3.8. Gene Prediction and Annotation

**Figure 6:**
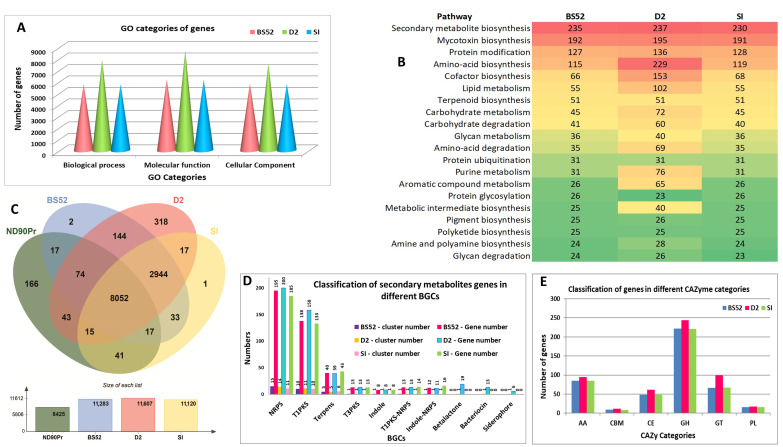
Gene prediction and annotation of three *B. sorokiniana* isolates, BS52, D2, and SI. (**A**) Classification of genes of the three isolates (BS52, D2, and SI) in different gene ontology (GO) categories. (**B**) Top 20 pathway predictions in BS52, D2, and SI isolates. (**C**) Orthologous clusters shared between the *B. sorokiniana* strain ND90Pr and three *B. sorokiniana* isolates BS52, D2, and SI. (**D**) Gene classification in different Biosynthetic Gene Clusters (BGCs) of secondary metabolites. (**E**) Classification of genes in different CAZyme categories.

The gene numbers predicted in BS52, D2, and SI were 11,516, 15,452, and 11,351, respectively (Table 1). The genes were classified into three gene ontology (GO) categories, cellular component, biological process, and molecular function. For BS52, the numbers of genes classified into three GO categories were cellular component (5812), biological process (5763), and molecular function (6186) (Table S4). For D2, the numbers of genes classified were cellular component (7553), biological process (7909), and molecular function (8693) (Table S5). Similarly, for SI, the numbers of genes classified were cellular component (5783), biological process (5746), and molecular function (6142) (Table S6 and Figure 6A). The predicted genes were categorized in various pathways in BS52, D2, and SI isolates (Table S7 and Figure 6B).

### 3.9. Gene Orthology

Orthologous gene clusters were identified among the *B. sorokiniana* strain ND90Pr, and the three isolates BS52, D2, and SI using OrthoVenn2 (Figure 6C). Proteins from these four *B. sorokiniana* strains/isolates formed 11,884 clusters. The reference *B. sorokiniana* strain ND90Pr formed 8425 clusters, while the three isolates BS52, D2, and SI formed 11,283, 11,607, and 11,120, respectively. A total of 8052 clusters were shared among all the four isolates/strains of *B. sorokiniana*. These commonly shared clusters showed GO-enriched terms for the biological process of amino acid transport (GO:0006865) and molecular function of transferase activity (transferring acyl groups) (GO:0016746). The reference *B. sorokiniana* strain ND90Pr comprised 166 unique clusters representing 478 predicted proteins, while 2, 318, and 1 cluster representing 4, 1041, and 2 predicted proteins were unique to BS52, D2, and SI, respectively. Approximately 2.739% of the D2 cluster was found to be unique, which was higher compared to unique clusters observed for BS52 (0.017%), SI (0.008%), and the reference strain ND90Pr (1.970%). Within the 318 unique clusters of D2, the GO-enriched terms for biological processes included amino acid transport (GO:0006865), regulation of transcription (GO:0006355), protein secretion by the type II secretion system (GO:0015628), DNA-templated transcription and initiation (GO:0006352). Similarly, the GO-enriched terms for molecular function included sequence-specific DNA binding transcription factor activity (GO:0003700) and symporter activity (GO:0015293).

### 3.10. Prediction of COGs

Prediction of the Clusters of Orthologous Groups (COGs) was performed using EggNOG v5.0 [37]. For BS52, the functions of 11,457 unigenes annotated in the COG database were predicted and classified into 23 categories (Table S8). The largest group was ‘carbohydrate transport and metabolism’, representing 12.6% of the COG-annotated unigenes. It was followed by ‘post-translational modification, protein turnover, and chaperones’ (8.92%), ‘signal transduction mechanism’ (8.01%), and others (Table S9 and Figure S3A). Similarly, for D2, the functions of 15,216 unigenes annotated in the COG database were predicted and classified into 23 categories (Table S10). The largest group was ‘carbohydrate transport and metabolism’, representing 11.20% of the COG-annotated unigenes. It was followed by ‘post-translational modification, protein turnover, and chaperones’ (7.94%), ‘transcription’ (7.71%), and others (Table S11 and Figure S3B). Likewise, for SI, the functions of 11,327 unigenes annotated in the COG database were predicted and classified into 23 categories (Table S12). In this case, too, the largest group was ‘carbohydrate transport and metabolism’, representing 12.70% of the COG-annotated unigenes, which was again followed by ‘post-translational modification, protein turnover, and chaperones’ (8.88%), ‘signal transduction mechanism’ (7.98%), and others (Table S13 and Figure S3C). In the ‘defense mechanisms’ category, a high number of genes, i.e., 99 (0.97%), were found in the isolate D2 and a comparatively low number of genes, i.e., 49 each (0.66%), were observed in the isolates BS52 and SI.

### 3.11. Prediction of BGGs

Biosynthetic gene clusters (BGCs) are the organized groups of genes involved in the synthesis of secondary metabolites. Prediction of BGCs was performed using antiSMASH v.5.0 [38]. For BS52, 34 BGCs were predicted, which included 15 non-ribosomal peptide synthetase (NRPS) clusters, 10 type-1 polyketide synthase (T1PKS), 5 terpenes, and 1 cluster each of type-3 polyketide synthase (T3PKS), indole, type-1 polyketide synthase (T1PKS)-NRPS hybrid, and indole-NRPS hybrid. For D2, 37 BGCs were predicted, which included 14 non-ribosomal peptide synthetase (NRPS) clusters, 11 type-1 polyketide synthase (T1PKS), 5 terpenes, and 1 cluster each of type-3 polyketide synthase (T3PKS), indole, type-1 polyketide synthase (T1PKS)-NRPS hybrid, indole-NRPS hybrid, beta-lactone, bacteriocin, and siderophore. Similarly, for SI, 31 BGCs were predicted, which comprised 11 non-ribosomal peptide synthetase (NRPS) clusters, 10 type-1 polyketide synthase (T1PKS), 6 terpenes, and 1 cluster each of type-3 polyketide synthase (T3PKS), indole, type-1 polyketide synthase (T1PKS)-NRPS hybrid, and indole-NRPS hybrid (Figure 6D).

### 3.12. Prediction of CAZymes

It was found that 441, 520, and 441 genes were annotated as CAZymes using the CAZy database for BS52, D2, and SI, respectively. Interestingly, some genes were annotated under two or more classes simultaneously. These CAZymes could be divided into five categories and one structural domain. In BS52, 85 genes were annotated as Auxiliary Activities enzymes (AAs), 48 genes as Carbohydrate Esterases (CEs), 222 genes as Glycoside Hydrolases (GHs), etc. Similarly, for D2, 95 genes were annotated as Auxiliary Activities enzymes (AAs), 61 genes as Carbohydrate Esterases (CEs), 244 genes as Glycoside Hydrolases (GHs), etc. Likewise, for SI, 85 genes were annotated as Auxiliary Activities enzymes (AAs), 48 genes as Carbohydrate Esterases (CEs), 221 genes as Glycoside Hydrolases (GHs), etc. (Table S14 and Figure 6E).

### 3.13. Prediction of Secretory Proteins

Secreted proteins were predicted using SignalP. The predicted secretome for D2 was relatively large, comprising 1022 secretory proteins. In comparison, the isolates BS52 and SI had only 620 and 618 secretory proteins (Tables S15–S17). The secretome was made up of 8.74%, 10.19%, and 8.77% of the predicted proteomes for BS52, D2, and SI, respectively. Several genes encoding secretory proteins were unique in the isolate D2. The unique genes, CUTI1, PME, CATB, FCK1, RED3, etc., were found to be involved in molecular functions, such as cutinase activity, pectinesterase activity, catalase activity, cytokinin biosynthesis, alcohol dehydrogenase (NAD+) activity, etc.

### 3.14. Prediction of Pathogenicity Genes

Fungal pathogenicity genes were annotated using the Pathogen–Host Interaction (PHI) database. A total of 2015, 2476, and 2018 putative pathogenicity genes were annotated in BS52, D2, and SI, respectively (Tables S18–S20). The major group of genes belonged to the classes transcription factor, protein kinase, ABC transporter, hypothetical protein, and polyketide synthase.

The phytopathogenic genes, FZC65 and VdSso1, were only found in the isolate BS52. Similarly, the phytopathogenic genes viz., GzHOME009, ACC deaminase (VDAG_10392), GzOB039, MoHYR1, HopI1, PKS9, KSA1, GzWing026, and OXI1 were specifically found in the isolate D2; while the phytopathogenic genes AaGa1, GzHOMEL026, GzGRF, GzZC111, GzHMG003, Fgsg11343, GzAT001, MGG_04582, and VdCYP1 were found only in the isolate SI. These genes are known to be present in pathogenic fungi infecting crop plants (Table S21).

**Figure 7:**
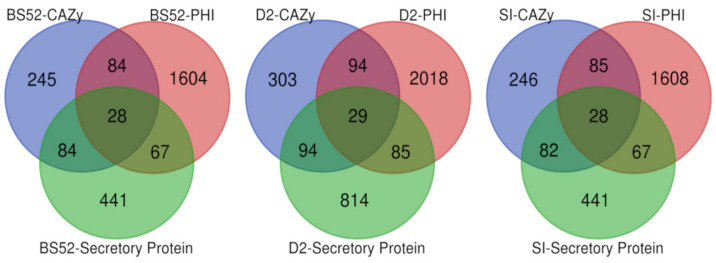
Venn diagram showing the overlap of annotations in CAZy, PHI, and Signal P (secretory proteins) databases in BS52, D2, and SI isolates.

The overlap of common genes predicted for CAZy, secretory protein, and PHI in the isolates BS52, D2, and SI was drawn using a Venn diagram. A total of 28, 29, and 28 common genes formed an overlap among the CAZy, PHI, and secretory proteins in the isolates BS52, D2, and SI (Figure 7).

### 3.15. Prediction of Target Genes in D2 Isolate

**Figure 8:**
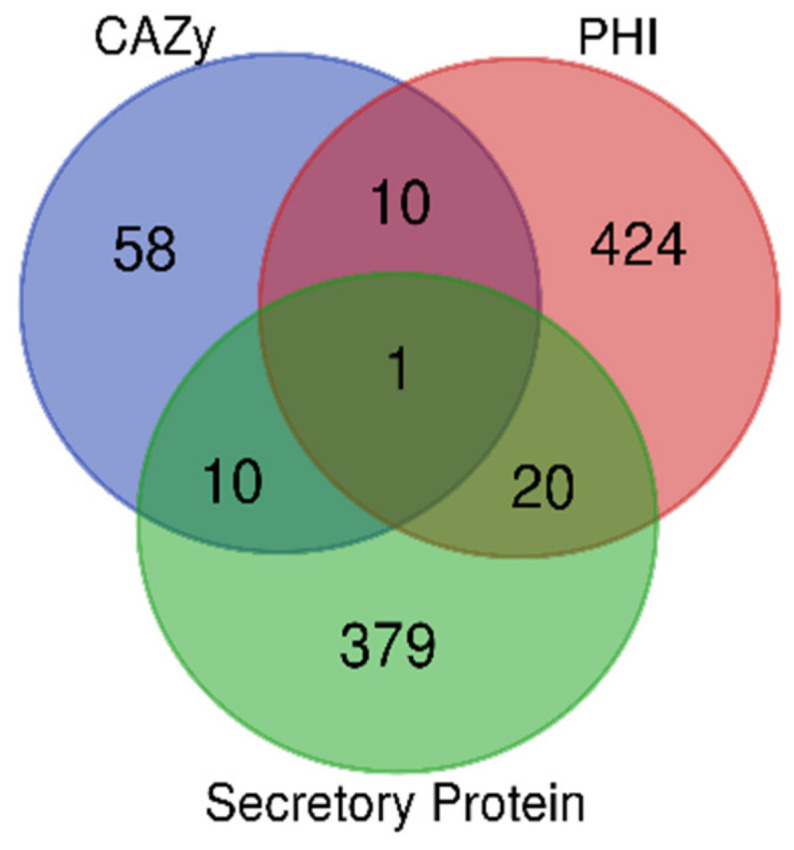
Venn diagram showing the overlap of annotations in CAZy, PHI, and Signal P (secretory proteins) databases among the unique genes found in the D2 isolate.

A total of 4069 genes were uniquely detected in the D2 isolate. Functional prediction of these genes revealed 79, 455, and 410 genes representing CAZy, PHI, and secretory proteins, respectively. However, only 1 gene was found to be common among all three categories (Id-CDS000012722, gene annotated-MLTD). Moreover, 10 genes were common between CAZy and PHI, and also between CAZy and secretory proteins. Similarly, 20 genes were common between PHI and secretory proteins. These genes could be further evaluated as potential targets for pathogen control (Table S22 and Figure 8).

## 4. Discussion

Like many phytopathogenic fungi, *B. sorokiniana* also exhibits high morphological and pathological variations [42]. In the present study, we observed morphological variability among the isolates of *B. sorokiniana*. Of the 7 isolates, 3 (A, L, and HD3069) had similar morphology and white mycelia with similar melanization on PDA. However, these isolates varied in their growth patterns. The isolates BS52 and D2 had similar growth patterns on PDA but showed differences in melanization. In an earlier study [43], no correlation was observed between the genetic similarity of groups and geographical origins of *B. sorokiniana* isolates, inferring that the morphological characteristics are not conditioned solely by genetic composition. As isolates from the same species differ in their morphology and genetic compositions, this variability must be attributed to interactions between genetic and environmental conditions, such as ecology and climatic variations in the areas from where the isolates have been obtained.

Variation in environmental conditions, production of toxins, and other metabolites are among the few conditions that often result in variation in pathogenicity [23]. Studies that characterized pathogenic variations among the *B. sorokiniana* isolates from distinct geographical areas showed significant differences in host response [44]. In the present study, the pathogenicity of 12 *B. sorokiniana* isolates collected from three geographical regions of India was assessed on the wheat variety DDK 1025. Based on this, the 3 most virulent isolates (BS52, D2, and SI) were selected, which incidentally belonged to the three geographical regions. Among them, D2 was the most virulent. Milus et al. [45] also observed significant differences in aggressiveness and infection efficiency of 2 *B. sorokiniana* isolates belonging to the same pathotype. Sultana et al. [46] performed a similar investigation where 169 isolates of *B. sorokiniana* were assessed for pathogenic variability on the spot blotch-susceptible variety ‘Kanchan.’ They found a positive relationship between pathogenic variability and aggressiveness with agroclimatic conditions.

Genome sequencing and assembly of 3 isolates of *B. sorokiniana*, BS52, D2, and SI, revealed genome sizes of 35.19 MB, 39.32 MB, and 32.76 MB, respectively. The genome assembly of BS52, D2, and SI showed N50 lengths of 1054399, 803483, and 1063709 and GC contents of 48.48%, 50.43%, and 49.42%, respectively. The genome of the isolate D2 was found to be comparatively larger than that of BS52 and SI. Similar efforts to sequence the genomes of the pathogenic fungal isolates collected from different regions showed variation in genome size. The genome assembly of the 19 isolates of the fungal wheat pathogen *Zymoseptoria tritici* ranged from 37.13 Mb to 41.76 Mb [47]. Similarly, 2 isolates of the oat crown rust pathogen, *Puccinia coronata* f.sp. *avenae,* showed genome assembly of 99.16 Mb (12SD80) and 105.25 Mb (12NC29) [48]. It has been reported that a larger genome is more likely to contain a high number of secondary metabolite-related genes, virulence genes, and stress-tolerance genes [49].

BUSCO was used to assess the completeness of genome assembly utilizing a set of core single-copy orthologous genes [27]. The BUSCO alignment showed that final assemblies of BS52, D2, and SI were complete to approximately 99.08%, 99.06%, and 90.87%, respectively. The BUSCO prediction with >90% completeness indicated coverage of most of the genomic sequence space [16]. A phylogenetic tree constructed based on the single-copy orthologs of BS52, D2, and SI, with the other 11 genome sequences inferred that the isolates BS52, D2, and SI formed a clade with the 4 *B. sorokiniana* strains, indicating their genetic relatedness. Further, synteny analysis showed that all 3 *B. sorokiniana* isolates share a high degree of genomic synteny with the reference genome *B. sorokiniana* strain ND90Pr.

Orthologous gene clusters were identified between the *B. sorokiniana* strain ND90Pr, and the 3 isolates using OrthoVenn2. The isolates BS52, D2, and SI formed a large number of common clusters along with the reference strain ND90Pr. Among the 3 isolates, D2 showed several unique clusters (318) containing the genes involved in essential activities, such as sequence-specific DNA binding transcription factor activity, transcription regulation, and protein secretion, which might contribute to the increased pathogenicity of D2. The predicted secretome for D2 was also relatively large compared to that of the isolates BS52 and SI. The secreted proteins are known to impart virulence characteristics to phytopathogenic fungi. During plant–fungi interaction, the phytopathogenic fungi secrete several proteins, which play a crucial role in fungal penetration, colonization, and lesion formation [49]; thus, imparting higher virulence to the phytopathogenic fungi.

The Cluster of Orthologous Groups (COGs) results revealed that the genes for defense mechanisms were higher in the isolate D2 than BS52 and SI. Prediction of Biosynthetic gene clusters (BGCs) involved in secondary metabolite synthesis showed the highest number of genes for NRPS and PKS clusters in the D2 isolate. An expansion of NRPS and PKS backbone genes leads to increased production of secondary metabolites of these groups, which may play a significant role in host invasion by enabling appressorial penetration and pathogenicity [50]. The analysis of BGCs among the 3 *B. sorokiniana* isolates revealed that the beta-lactone, bacteriocin, and siderophore clusters were exclusively present in D2. These secondary metabolites are reported to impart increased virulence in fungal pathogens [51].

Phytopathogenic fungi use carbohydrate-active enzymes (CAZymes) to break down the cell wall and enter plant cells. CAZymes play a vital role during early infection by sequestering the chitin oligomers released by the fungus and preventing their recognition by the plant immune system [52]. Among the six categories of CAZymes, glycosyl hydrolases (GH), which efficiently break down the plant cell wall for penetration and successful infection [39], were highly abundant in all 3 isolates. However, D2 showed a high number of CAZymes in all six categories compared with isolates BS52 and SI, indicating the expansion of specific gene classes. The results were consistent with previous studies in *Colletotrichum* species that showed the expansion of specific gene classes potentially involved in pathogenesis, particularly CAZymes and secondary metabolites [50,53].

The prediction of secretome revealed that the number of secretory proteins was relatively large in isolate D2 compared with BS52 and SI. Several genes encoding secretory proteins such as CUTI1, PME, CATB, FCK1, RED3, etc. were unique in the isolate D2. These genes were enriched in gene ontologies, such as pathogenesis, cellular responses to hydrogen peroxide, hydrogen peroxide catabolic process, oxidative stress, invasive growth, in response to glucose limitation, pseudohyphal growth, etc. Similarly, these D2-specific genes were enriched in various pathways, including secondary metabolite biosynthesis, mycotoxin biosynthesis, polyketide biosynthesis, glycan metabolism, pectin degradation, etc. According to previous studies [Lu et al. [54] (cutinase activity), Sella et al. [55] (pectinesterase activity), Hernandez et al. [56] (catalase activity), Sørenson et al. [57] (cytokinin biosynthesis), Inderbitzin et al. [58] (alcohol dehydrogenase (NAD+) activity-mycotoxin production), etc.], the gene ontology and pathways of these D2-specific genes indicate their roles in fungal virulence, which could have contributed to D2 being more virulent than the isolates BS52 and SI.

The Pathogen–Host Interactions database (PHI-base) is a catalog of experimentally validated pathogenicity genes from plant and animal pathogens that are manually curated from peer-reviewed publications [41]. Most of the genes from *B. sorokiniana* isolates had homology to the genes from *Fusarium oxysporum*, *Magnaporthe oryzae*, and *Aspergillus fumigatus,* likely due to the extensive research carried out on these model fungi, leading to the highest represented pathogens in PHI-base. The comparative analysis of the PHI-base revealed more pathogenicity genes in the isolate D2 (2476), followed by SI (2018) and BS52 (2015). Additionally, the unique phytopathogenic genes were identified in each of the isolates.

Since the isolate D2 showed higher virulence than BS52 and SI, it was further analyzed to understand the possible reasons for its higher virulence. A total of 4069 genes were found to be unique in the D2 isolate. Among these, 41 genes were common in at least two of the three functional categories (CAZy, PHI, and secretory proteins), highlighting their importance in virulence. Among these, the following genes were reported to be associated with fungal pathogenicity, CDS_000012722 (cell wall organization and lytic transglycosylase) [59,60], CDS_000014640 (trehalose biosynthesis) [61], CDS_000011491 (acetylglucosaminyl transferase activity) [62], CDS_000012002 (lipid A biosynthesis) [63], CDS_000013078 (polygalacturonase activity) [64], and CDS_000012490 (glutathione metabolism) [65]. Functional validation of these genes will confirm their roles in pathogenicity and the molecular basis of variation in virulence of the 3 isolates of *B. sorokiniana*. The validated pathogenicity-related genes could be used as key targets to develop strategies to control the fungal pathogen.

The isolate D2 exhibited a relatively larger genome with expanded arsenals of BGCs, CAZymes, pathogenicity genes, and secretome, which might be responsible for imparting the higher pathogenicity relative to the isolates BS52 and SI. These results are consistent with the fungal pathogenicity assay where the isolate D2 caused more severe disease symptoms on wheat leaves than SI and BS52. Thus, our results provide valuable genomic data to further characterize the molecular mechanisms for increased virulence in *B. sorokiniana* isolates.

## 5. Conclusions

We sequenced three isolates of *B. sorokiniana*, BS52, D2, and SI, collected from three different geographical locations in India. These isolates showed similar growth patterns but differed in the levels of melanization, aggressiveness, and pathogenicity. Pathogenicity assessment revealed that the isolate D2 was more virulent than BS52 and SI. The whole-genome sequencing and de novo assembly resulted in genome sizes of 35.19 MB, 39.32 MB, and 32.76 MB for BS52, D2, and SI. The prediction of COGs, BGCs, CAZyme, secretome, and PHI-base revealed the expansion of gene classes involved in pathogenesis in the isolate D2, which might be responsible for imparting higher pathogenicity to D2 compared to the isolates BS52 and SI. This study provides the first comparative genome analysis of Indian isolates of *B. sorokiniana* based on whole-genome sequencing. This study also provides high-quality genome sequences and annotated genes with putative roles in pathogenicity. These genomic resources could be further explored through molecular, functional, and evolutionary analyses to develop novel disease control measures for this agronomically important wheat spot blotch pathogen.

## Figures and Tables

**Table 1:** Results of the assemblies and comparison among the three isolates.

Isolate	BS52	D2	SI
Total number of bases	35,194,358	39,322,362	32,762,667
Number of scaffolds	280	317	319
Minimum sequence length	962	966	893
Maximum sequence length	2,403,846	2,565,778	2,417,524
Average sequence length	125,246.83	123,655.23	102,383.33
N50 length	1,054,399	803,483	1,063,709
% (A + T)	49.31	47.19	49.16
% (G + C)	48.48	50.43	49.42
Number of genes predicted	11516	15452	11351

## Data Availability

All the raw sequence data are available via GenBank under the SRA accessions SRR17117508 (isolate BS52), SRR17117509 (isolate D2), SRR17117510 (isolate SI), and SRR17172563 (mate-pair sequence of D2). This whole-genome sequencing project of Indian isolates of wheat spot blotch pathogen *Bipolaris sorokiniana* has been deposited at NCBI GenBank under Submission IDs SUB10748268, BioProject ID PRJNA785882, comprising the BioSample IDs SAMN23588116 (BS52), SAMN23588117 (D2), and SAMN23588118 (SI).

## Supplementary Materials

The following supporting information can be downloaded at: https://www.mdpi.com/article/10.3390/pathogens12010001/s1, Table S1: Pathogenicity assay for the seven sporulating isolates; Table S2: Spot blotch disease scores on second leaf of wheat seedlings at 7 days post-inoculation of fungal isolates BS52, D2, and SI; Table S3: Disease severity of three Bipolaris sorokiniana isolates (BS52, D2, and SI); Table S4: Gene annotation information of BS52 isolate; Table S5: Gene annotation information of D2 isolate; Table S6: Gene annotation information of SI isolate; Table S7: Uniprot database hits for various pathways in BS52, D2, and SI isolates; Table S8: Cluster of Orthologous Groups (COGs) category distribution in isolate BS52; Table S9: Summary of Cluster of Orthologous Groups (COGs) category description of isolate BS52; Table S10: Cluster of Orthologous Groups (COGs) category distribution in isolate D2; Table S11: Summary of Cluster of Orthologous Groups (COGs) category description of isolate D2; Table S12: Cluster of Orthologous Groups (COGs) category distribution in isolate SI; Table S13: Summary of Cluster of Orthologous Groups (COGs) category description of isolate SI; Table S14: Carbohydrate-active enzymes (CAZYmes) in three Bipolaris sorokiniana isolates BS52, D2, and SI; Table S15: Predicted secretome of isolate BS52; Table S16: Predicted secretome of isolate D2; Table S17: Predicted secretome of isolate SI; Table S18: Gene annotation of isolate BS52 in Pathogen-Host Interaction (PHI) database; Table S19: Gene annotation of isolate D2 in Pathogen-Host Interaction (PHI) database; Table S20: Gene annotation of isolate SI in Pathogen-Host Interaction (PHI) database; Table S21: Unique fungal pathogenicity genes in three fungal isolates based on Pathogen-Host Interaction (PHI) database; Table S22: Unique target genes in the D2 isolate based on CAZy, Sectretory proteins, and PHI database.
