# Supplementary material for: Whole Genome Sequencing and Comparative Genomics of Indian Isolates of Wheat Spot Blotch Pathogen Bipolaris sorokiniana Reveals Expansion of Pathogenicity Gene Clusters
Source: Pathogens. 2022 Dec 20;12(1):1. doi: 10.3390/pathogens12010001 (PMC9865733; doi:10.3390/pathogens12010001)
Supplement: Supplementary file 1 [file pathogens-12-00001-s001.zip › pathogens-1984259-supplementary-Figure.pdf]

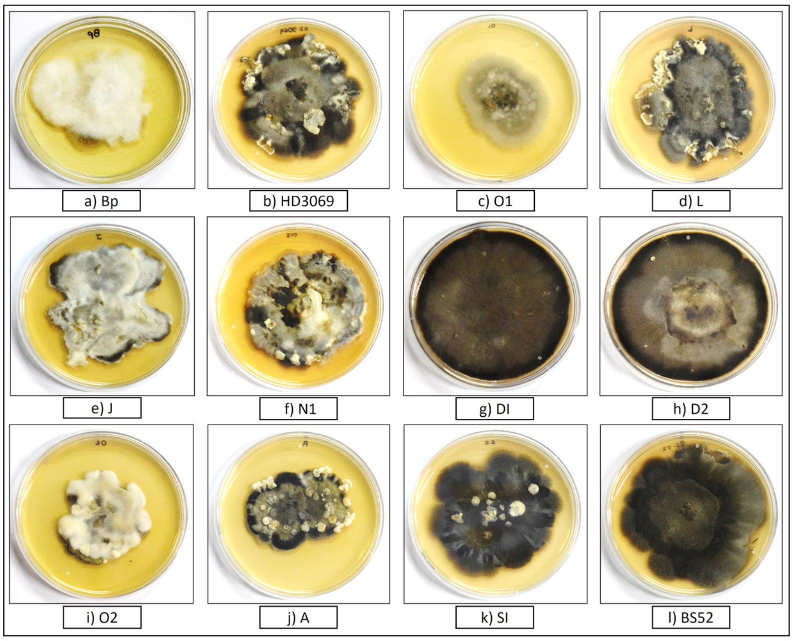

Figure S1: Morphology of isolates of *B. sorokiniana* collected from different geographical regions of India and grown on PDA plates

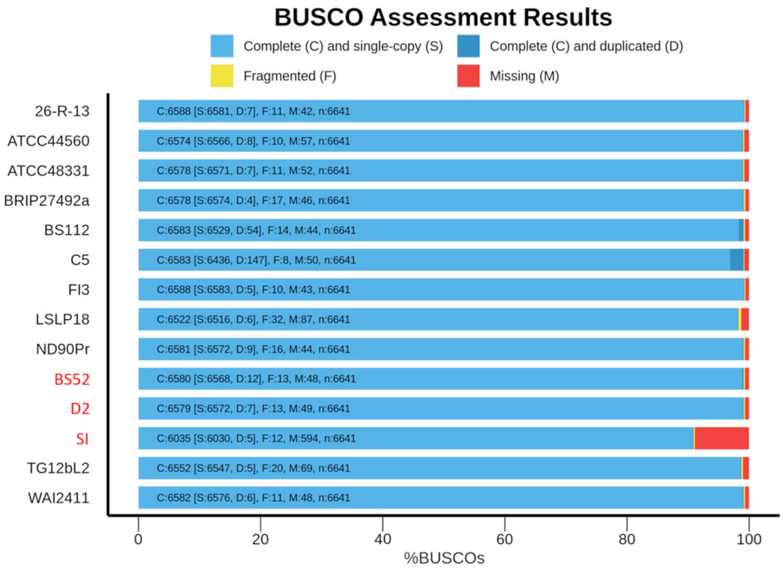

Figure S2: BUSCO assessment results of genome completeness of BS52, D2, and SI compared to 11 genome sequences, including seven strains belonging to five *Bipolaris* species (26-R-13 (*B. zeicola*), ATCC44560 (*B. oryzae*), ATCC48331 (*B. maydis*), C5 (*B. maydis*), FI3 (*B. victoriae*), LSLP18 (*B. cookei*), and TG12bL2 (*B. oryzae*)) and four *Bipolaris sorokiniana* strains (BRIP27492A, BS112, ND90Pr, and WAI2411)

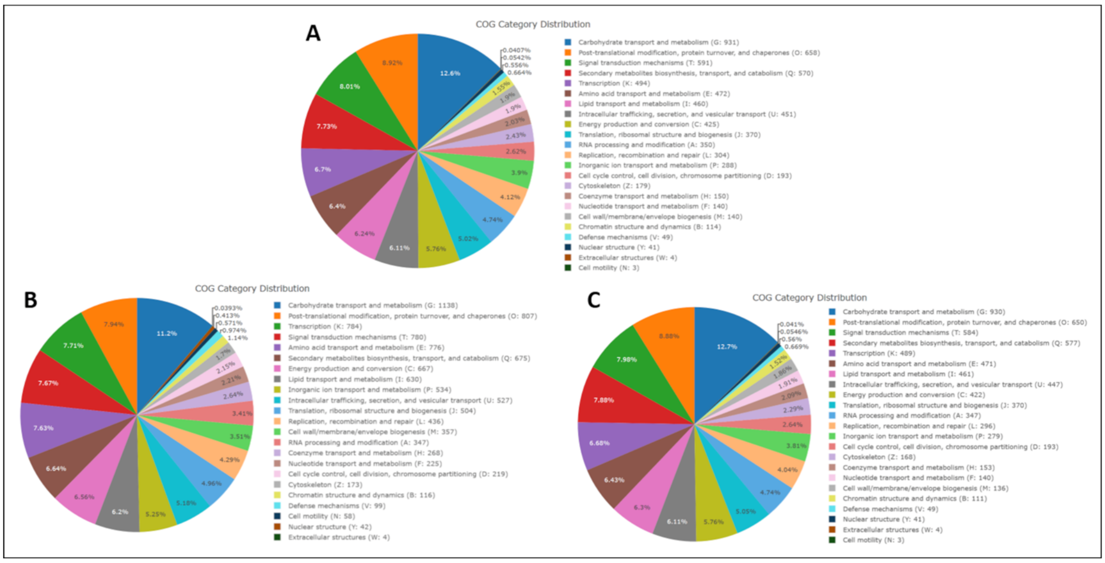

Figure S3: COGs category distribution in isolates (A) BS52, (B) D2, and (C) SI
